# Revisiting Planting Density in Rainfed Olive: Yield, Water Productivity and Resilience in Different Mediterranean Regions

**DOI:** 10.3390/plants15172651

**Published:** 2026-08-29

**Authors:** Omar Garcia-Tejera, Álvaro López-Bernal

**Affiliations:** 1 Departamento de Ingeniería Agraria y del Medio Natural, Universidad de La Laguna, 38296 San Cristobal de La Laguna, Spain; 2 Departamento de Agronomía, Universidad de Córdoba, Campus de Rabanales, Edificio C4, 14071 Córdoba, Spain; g42lobea@uco.es

**Keywords:** water-use efficiency, soil evaporation, drought, soil depth, Finlay–Wilkinson stability

## Abstract

Across the Mediterranean, most olive orchards are still rainfed and have traditionally been planted at low density on the assumption that wide spacing buffers the risk of crop failure in dry years. We tested this assumption with the process-based model OliveCan, simulating three planting densities (100, 204 and 408 trees ha^−1^) at three contrasting sites (Córdoba, Izmir and Pisa) on shallow and deep soils under a baseline climate and two perturbations (+2 °C and −10% rainfall). Increasing density raised both yield and water productivity (*WP_y_*, the yield-to-evapotranspiration ratio) in all studied scenarios. As density increases, soil evaporation (*E_s_*) and runoff are reduced, and more water can be used for transpiration (*E_p_*). Our analysis suggests that yield gains overcome *E_p_* rises when tree density increases, resulting in a better *WP_y_*. Deep soils out-yielded shallow ones and buffered climatic stress. Yields were robust to a moderate, evenly distributed rainfall reduction but were eroded by warming. A stability analysis showed the highest density to be the most productive across the studied site × climate scenario × simulation year × soil depth combinations, while the lowest planting density was more stable. The present work explores new possibilities for rainfed olive farmers. Fieldwork would help test the results presented here and tailor new tree densities for rainfed olive orchards.

## 1. Introduction

With more than 8 million hectares, the olive (*Olea europaea* L.) is the defining tree crop of the Mediterranean basin, with major production concentrated in five countries: Greece, Italy, Spain, Tunisia and Turkey [1]. The great majority of this area is still managed under rainfed, low-density conditions in which water, rather than radiation or nutrients, ultimately limits production. Traditional dryland orchards are planted at low densities (≤100 trees ha^−1^) [2], reflecting a long-standing and conservative grower rationale. Allocating a large volume of soil to each tree is assumed to lower the risk of crop failure in dry years by enlarging the per-tree soil-water reservoir. This logic, however, has rarely been tested against the productivity of denser plantings under rainfed conditions, and it is now being challenged on two fronts. Climate change and growing water scarcity are projected to raise evaporative demand and intensify drought across the basin, threatening the sustainability of rainfed olive growing [3,4], while the rapid expansion of irrigated, high-density plantations is eroding the competitiveness of traditional systems and sharpening the need for rainfed management strategies that are both productive and resilient.

A substantial body of evidence shows that intensification (i.e., raising planting density) increases not only yield per unit land but also water productivity (*WP_y_*, yield-to-evapotranspiration ratio [5]) across a wide range of water regimes [6,7,8]. However, this work has been exclusively performed on irrigation trials. To the best of our knowledge, only one study has addressed the impact of intensification under rainfed conditions. Guerfel [9] showed that increasing density from 51 trees ha^−1^ to 156 trees ha^−1^ raised yield by 53%. The mechanism is related to evapotranspiration (*ET*) partitioning. At low canopy cover, a large share of total evapotranspiration is lost as direct soil evaporation, whereas denser canopies intercept more radiation, shade and cool the soil, and shift the partitioning from evaporation (*E_s_*) toward tree transpiration (*E_p_*) [10,11]. Because yield in water-limited environments scales closely with transpiration, reducing evaporative losses should in principle improve both *WP_y_* and yield.

Several gaps nonetheless keep the rainfed case unresolved. First, the evaporation-saving benefit of a denser canopy is offset to an unknown degree by intensified tree-to-tree competition for a finite soil-water store. Rainfed trials have shown stomatal and water potential penalties as tree density increases in arid climates [9]. Where the balance of this trade-off lies has yet to be quantified. Second, soil depth—the very factor underlying the grower’s conservative spacing—has scarcely been studied as an explicit resilience variable in olive; the relevant mechanistic evidence that deep rooting buffers drought comes largely from general ecohydrology [12,13], with the plasticity of olive root systems documented mainly under deficit irrigation [14]. Finally, the empirical experiments needed to address these questions are time- and land-consuming, since they would require well-established trees, multiple sites and many seasons [15]. As a result, the net impact of increasing density under rainfed conditions and whether the attendant *WP_y_* gain stems from higher yield, lower crop evapotranspiration, or both remain unclear.

Process-based simulation models offers a way around these constraints. Models can explore management strategies across many site–year–climate combinations at low cost and help tailor field experiments [15]. OliveCan, a process-based model of olive development, growth and yield that couples orchard water and carbon balances and has been validated across contrasting management and water regimes, is well suited to this task [16]. OliveCan has been previously used to explore olive sustainability under different climatic scenarios [17], compare traditional with new olive farming systems [18] or explore the impacts of cover crops on rainfed olive orchards [19]. Here, we use OliveCan to assess how different planting densities (100, 204 and 408 trees ha^−1^) affect yield and water use in rainfed olive orchards at three contrasting Mediterranean sites spanning a water-limited to sub-humid gradient (Córdoba, Spain; Izmir, Turkey; Pisa, Italy) on shallow and deep soils. To expand the range of environments, we compared the baseline climate against two independently imposed scenarios: 2 °C warming and a monthly 10% reduction in precipitation.

We test two linked hypotheses: (i) that a moderate increase in planting density under rainfed conditions improves *WP_y_* and (ii) that it does so while raising, rather than compromising, yield across environments.

## 2. Materials and Methods

### 2.1. Model Description

Simulations were performed using the OliveCan model [16]. OliveCan is a process-based model that allows the simulation of the development, growth and yield of olive orchards under different water regimes. The model has been tested for growth, *ET* and yield on a variety of management practices and water regimes.

In brief, the model simulates the water balance by dividing the soil horizontally into three soil compartments. Each soil compartment is subdivided into a number of layers defined by the user. Fluxes of precipitation, runoff, drainage, water redistribution, and root water uptake are computed for each soil compartment and layer. Soil evaporation and direct evaporation of rainwater intercepted by the canopy are also considered.

Gross photosynthesis is calculated with a combination of [20] and [21] models that make it correctly responsive to atmospheric CO_2_ concentration and to the stomatal behavior induced by water stress. The model computes the intercepted photosynthetically active radiation according to the canopy geometrical structure, leaf density and size. Water stress is considered by the model through the integration of the SPAC model developed in [22]. Growth and development of the tree and the carbon exchange of the orchard are accounted for by computing the different fluxes of C assimilation and respiration in the tree and soil. The model allocates assimilates to the growth and respiration of different organs (leaves, shoots, branches, coarse roots, fine roots and fruits). Flowering date is simulated according to the model of [23], which considers two stages for which their duration depends on temperature. The first stage corresponds to the accumulation of chilling units, enabling the tree buds to overcome dormancy, while the second stage requires a given thermal time to reach flowering. Fruit photosynthesis and remobilization of CO_2_ assimilate are also considered.

As inputs, the model requires soil water contents at field capacity, wilting point and saturation; the hydrological condition (an indicator of the capacity of infiltration of the soil when it is wet) according to the method of the Soil Conservation Service (US-SCS); bulk density; and pH. In addition, the initial values of water for each soil compartment and soil layer are to be defined. Orchard characteristics are specified in the model by setting the planting density; row orientation; latitude; and initial values of age, ground cover and leaf area density. The model runs on a daily time step. A detailed description of the model is given in [16].

### 2.2. Simulation Analysis

#### 2.2.1. Climatic Scenarios

Simulations were conducted at three sites with contrasting rainfall regimes and reference evapotranspiration (*ET*_0_): Córdoba (37.85° N, 4.85° W, Spain), Pisa (43.7° N, 10.4° E, Italy), and Izmir (38.43° N, 27.16° E, Turkey). These sites were selected because together they span a climatic gradient from dry to sub-humid conditions. Weather data were obtained from the stochastic weather generator ClimaSG [24]. We compared three different climatic scenarios: (a) a *Baseline* scenario; (b) *T* + 2 °C, where mean temperature was increased by 2 °C; and (c) *P* − 10%. *P* − 10% was imposed by reducing the mean monthly rainfall input by 10% in each of the twelve months. Because ClimaSG reconstructs daily rainfall from monthly statistics rather than rescaling an existing daily series, the perturbed series is consequently an independent stochastic realization rather than a scaled version of the *Baseline*. We generated 28 years of weather data. 

We deliberately held CO_2_ constant. Earlier work showed that rising CO_2_ can partially offset the increase in transpiration driven by warming [18]. Our aim, however, was not to reproduce a realistic future climate but to broaden the range of environments sampled by perturbing temperature or precipitation on the *Baseline* climate scenario.

#### 2.2.2. Management Scenarios

At each site, we simulated three planting densities, 100, 204, and 408 trees ha^−1^, which correspond to the densities evaluated in [8]. The 100 trees ha^−1^ density is representative of commercial dryland orchards across the Mediterranean, 204 trees ha^−1^ represents the limit between irrigated and rainfed systems, and 408 trees ha^−1^ is a widely used density in irrigated orchards [25]. At each planting density, we simulated two soil depth scenarios: shallow (0.60 m) and deep (2 m). The two depths were chosen as contrasting bounds rather than modal values.

Simulations were run in static mode, meaning that, every year, the trees’ state variables were reset to their initial values, including the number of potential fruiting positions. The water balance variables, however, were not. The simulations therefore did not reproduce the alternate bearing or carry-over effects of water or heat stress. The analysis thus tests whether the orchard water and carbon balance can sustain a higher planting density. Table 1 summarizes the initial orchard characteristics used in all simulations. For each site, we simulated annual yield; water productivity (*WP_y_*, yield-to-ET ratio) [5] and the different components of the water balance (*E_s_*, *E_p_*, drainage (*D*), runoff (*R*) and canopy rain interception (*E_i_*)); and the maximum transpiration in the absence of stress (*E_pmax_*). The soil was loamy-textured. All simulations were performed using the same cultivar (‘Picual’).

### 2.3. Stability Calculations

The sensitivity of each planting density to each combination of site, climate scenario, simulation year and soil depth was characterized by adapting the joint regression approach originally developed to assess genotype stability in plant-breeding programs to the combination of densities and climates [26,27]. The joint regression approach evaluates the performance of plant genotypes across a variety of environments by regressing the yield of each genotype against an environmental index (*EI*). In our analysis, each combination of site, climate scenario, simulation year and soil depth was treated as a distinct ‘environment’, and the *EI* of each environment was defined as the mean fresh yield across the three planting densities present in it. For each density, annual fresh yield was then regressed linearly on the *EI* across all environments (*yield* = *a* + *b*·*EI*). The slope (*b*) quantifies the responsiveness of a density to environmental quality: A value greater than unity denotes an above-average response, with disproportionately large yields in favorable environments but greater losses in unfavorable ones (lower stability); a value close to unity denotes average responsiveness; a value below unity denotes a buffered, more stable response. The regression was performed separately for each soil depth. All analyses were conducted in Python 3.14 using pandas, statsmodels and matplotlib libraries.

## 3. Results

### 3.1. Climatic Conditions

In the *Baseline* scenario, Pisa had the largest annual precipitation (936 mm) and the lowest annual *ET*_0_ (974 mm). Compared to Cordoba, Izmir was characterized by higher annual precipitation (786 vs. 671 mm) and *ET*_0_ (1403 vs. 1281 mm). At *T* + 2 °C, both maximum and minimum temperatures increased. Moreover, *ET*_0_ rose to 1411, 1597 and 1098 mm in Cordoba, Izmir and Pisa, respectively. At *P* − 10%, precipitation was reduced to 637 mm, 641 mm and 867 mm in Cordoba, Izmir and Pisa, respectively (Table 2).

### 3.2. Yield and Water Balance

**Figure 1:**
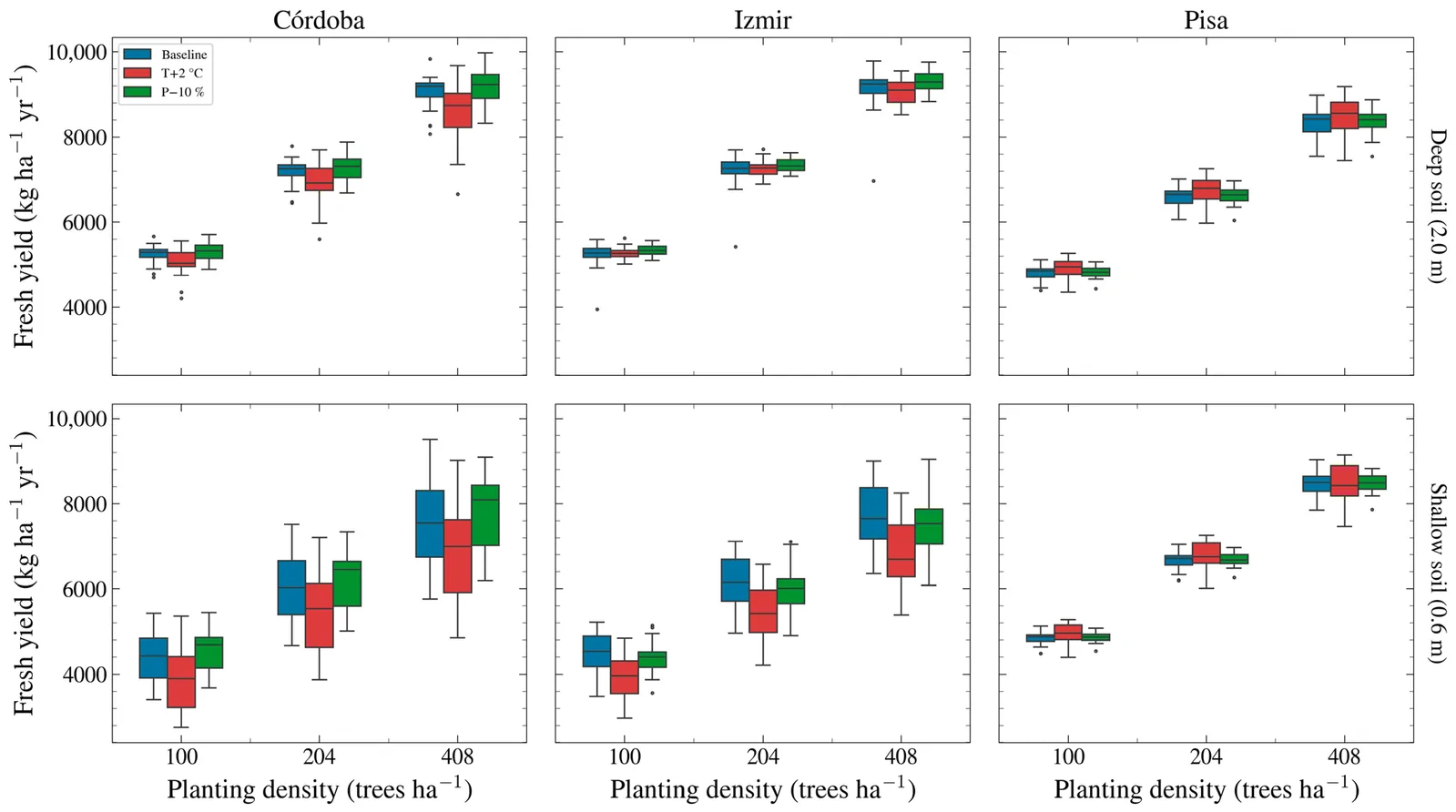
Boxplots of fresh yield by planting density and site for both soil depths: shallow (0.6 m) and deep (2 m). Box: Interquartile range; line: median; whiskers: 1.5× IQR; dots: outliers. Colors denote climate scenario (blue: *Baseline*; red: *T *+ 2 °C; green: *P* − 10%).

Median fresh yield increased progressively with planting density irrespective of the site, soil depth and climatic scenario (Figure 1). In the Córdoba *Baseline* scenario under deep soil, median yields increased from 5295 to 7258 kg ha^−1^ for 100 and 204 trees ha^−1^, respectively, and to 9189 kg ha^−1^ for 408 trees ha^−1^. This increasing trend was repeated in other locations, scenarios, and soil depths. Yield variability also increased with tree density across all simulated conditions. However, absolute variability differed by soil depth. In shallow soils, the yield standard deviation rose from 569.1 to 962.5 kg ha^−1^ when increasing density from 100 to 408 trees ha^−1^, whereas in deep soils, it increased from 290.7 to 548.6 kg ha^−1^ across the same density range. Yield variability was also affected by the location. Pisa presented the lowest variability regardless of the soil depth and the climatic scenario analyzed, whereas Cordoba had the highest variability.

Median yields for the *Baseline* and *P* − 10% scenarios were similar across all evaluated combinations, with only minor differences in yield variability. For instance, at a density of 408 trees ha^−1^ in Córdoba shallow soil, the median yield was 8062 trees ha^−1^ for the *Baseline* scenario and 8168 trees ha^−1^ for the *P* − 10% scenario, while the corresponding standard deviations were 841 and 756 trees ha^−1^, respectively. Conversely, the *T* + 2 °C scenario exhibited the lowest yields, except in Pisa (across all conditions) and in Izmir under the deep soil scenario.

Averaged across all 18 combinations of site, climate scenario, and soil depth, increasing density from 100 to 204 trees ha^−1^ increased the mean fresh yield by 37%, whereas increasing density from 100 to 408 trees ha^−1^ resulted in a 72% increase. This yield response was larger than the variability within any given density. Consequently, in every simulated environment, the effect of planting density exceeded the standard deviation of annual yield.

**Figure 2:**
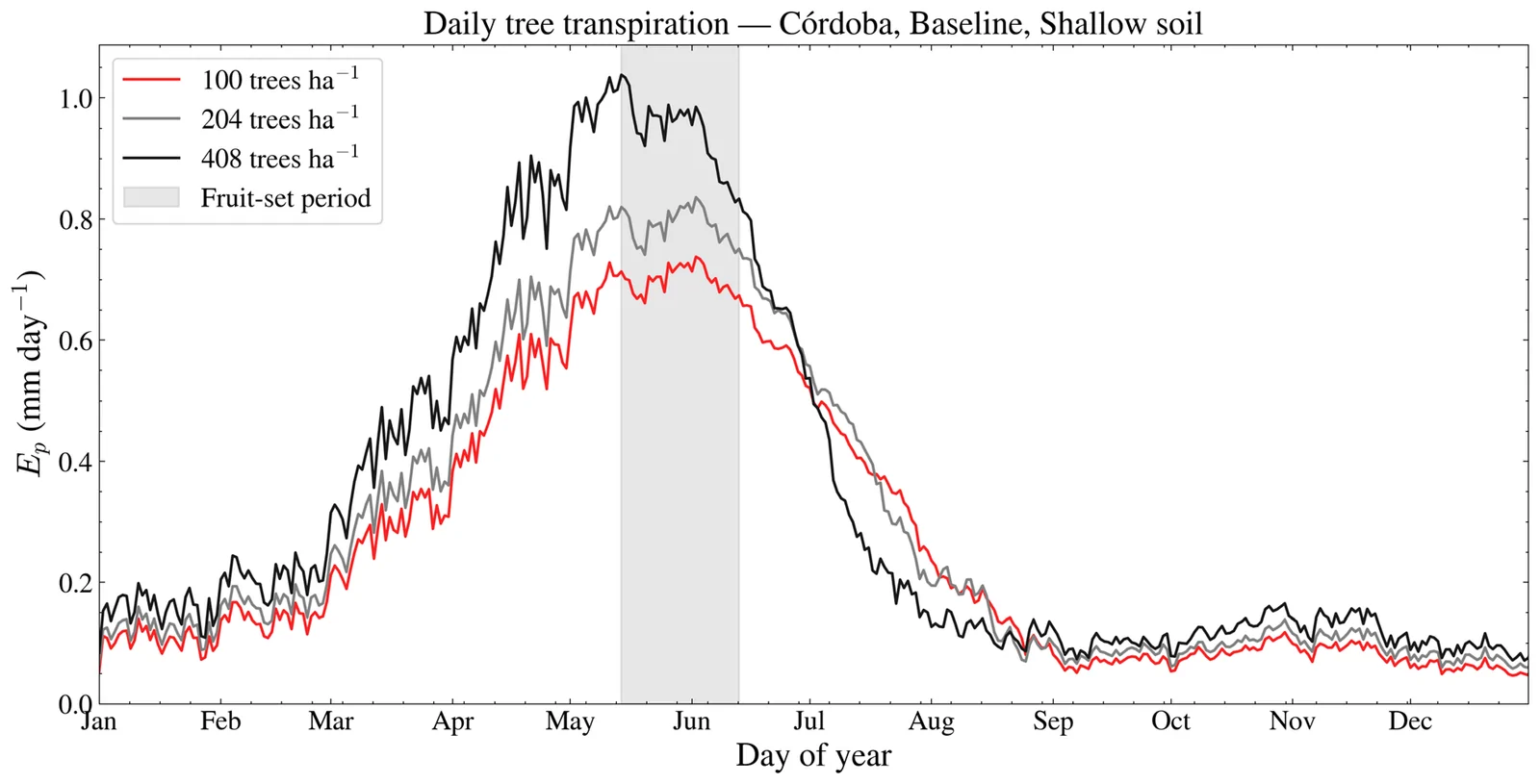
Mean daily tree transpiration (*E*_p_, mm day^−1^), averaged across all simulated years for Córdoba, *Baseline* scenario, shallow soil (0.6 m). Line colors denote planting density (red: 100 trees ha^−1^; grey: 204 trees ha^−1^; black: 408 trees ha^−1^). The shaded window (DOY 100–170, April–June) indicates the approximate period of fruit set.

The seasonal *E_p_* trends presented in Figure 2 provide an example of the difference among tree densities. *E_p_* rises from winter to spring and peaks around May–June, with the highest and lowest values found for 408 and 100 trees ha^−1^, respectively. This period coincides with fruit set (shadowed region in Figure 2). Afterwards, *E_p_* declines at different rates depending on tree density (i.e., the higher the density, the steeper the decline), transitorily reverting the ranking of *E_p_* rates during midsummer (i.e., *E_p_* was the lowest for 408 trees ha^−1^ in July and August). From September onwards, all densities presented similar *E_p_* rates, with the 408 trees ha^−1^ density slightly above.

Increasing planting density systematically altered the partitioning of precipitation among water balance components (Table 3 and Table 4). *E_s_* declined progressively with density as greater canopy cover intercepted more radiation and suppressed direct soil heating. *E_i_* (and so direct evaporation from the leaf surface) increased with density too. *E_i_* increase had a direct impact on runoff, which declined at higher densities because larger canopy interception reduces the amount of rainfall reaching the soil. *D*, however, followed a different pattern. Maximum drainage was found at 204 tree ha^−1^ at both soil depths. The same pattern was reproduced irrespective of the soil depth scenario.

**Figure 3:**
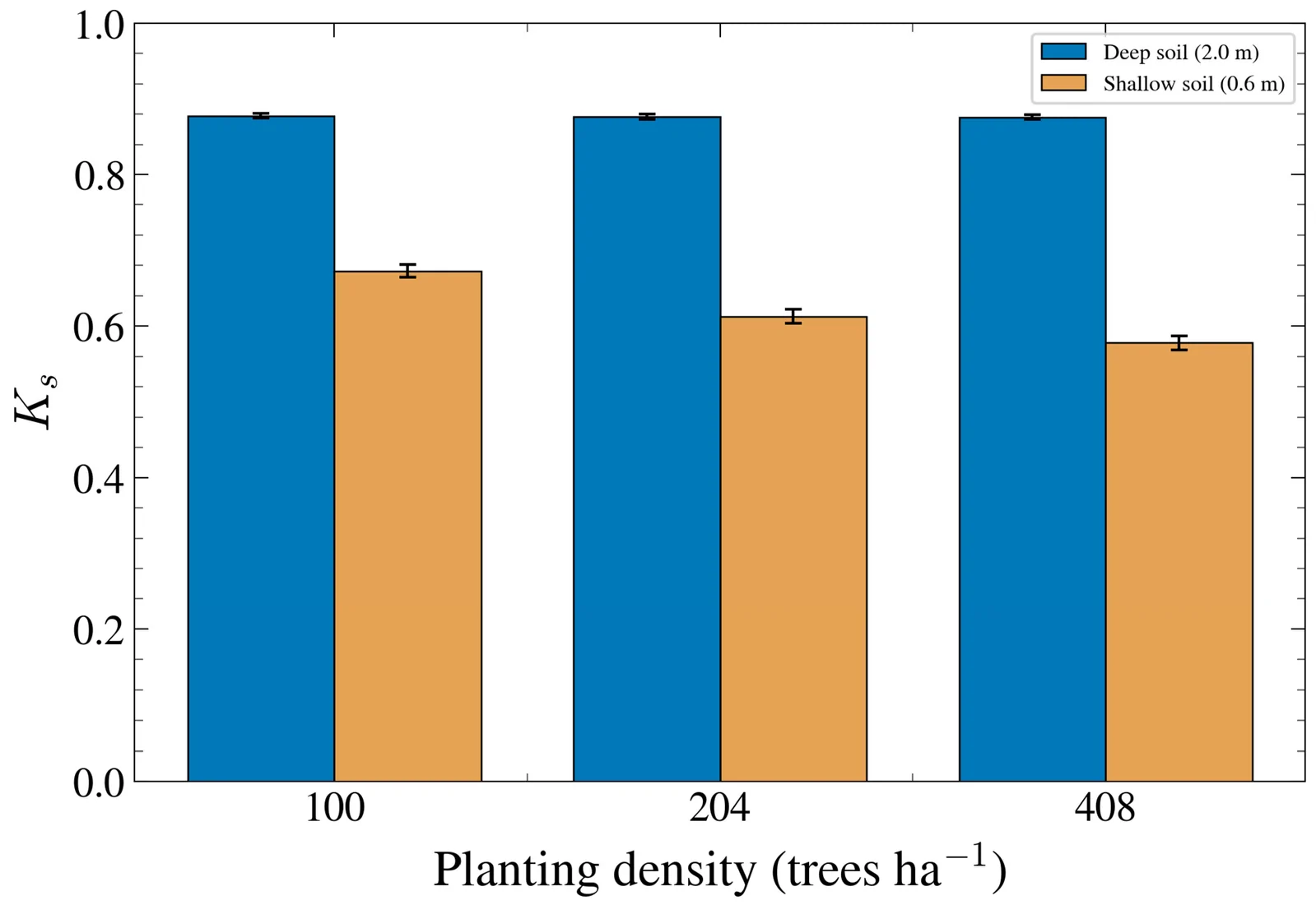
Mean seasonal water-stress coefficient (*K_s_* = *E_p_*/*E_pmax_*) by planting density on deep (2.0 m) and shallow (0.6 m) soils, averaged across the three sites, three climate scenarios and all years. Error bars are standard errors.

**Figure 4:**
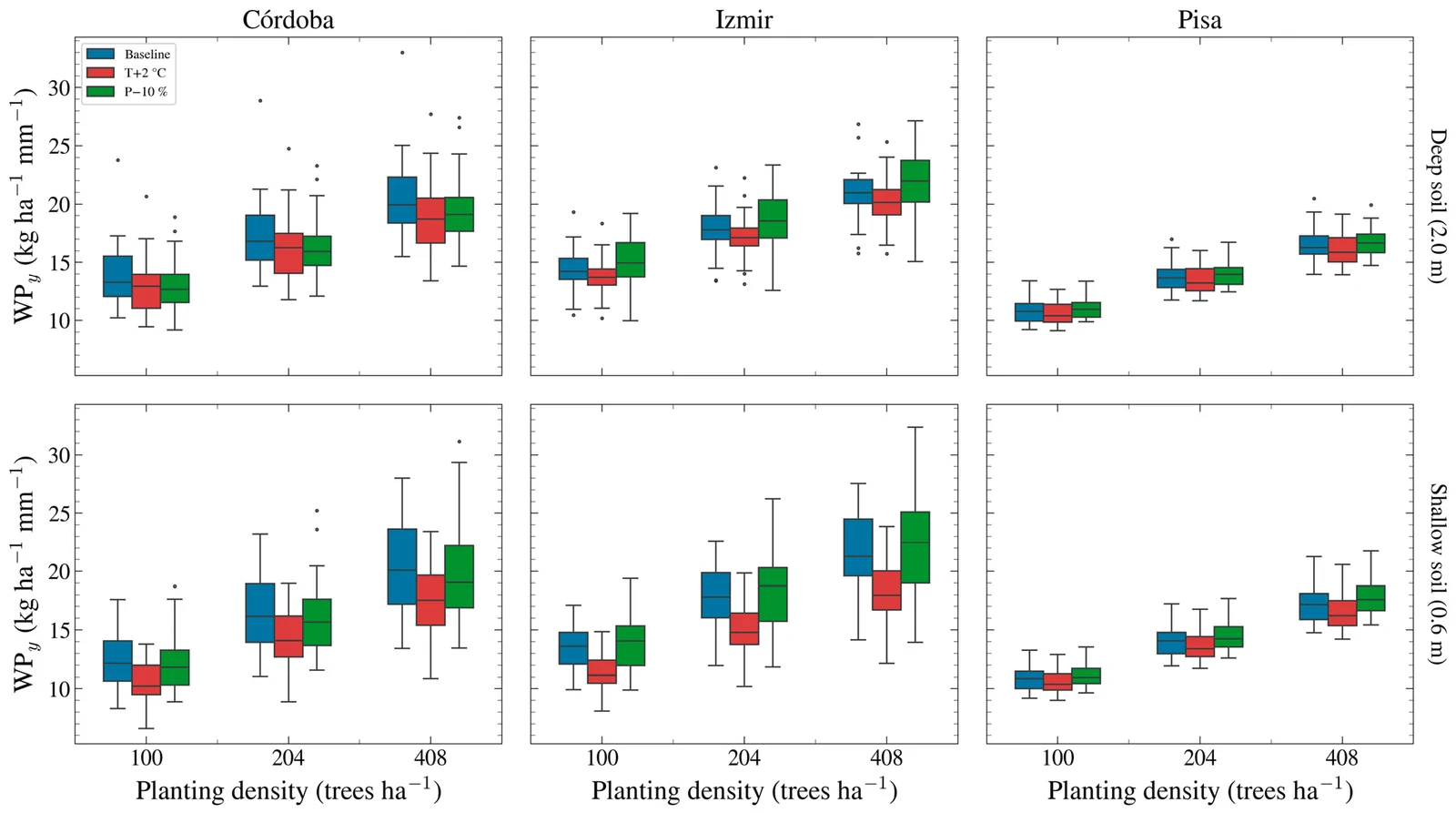
Boxplots of annual yield water use efficiency (*WP_y_*, kg ha^−1^ mm^−1^) by planting density and site for both soil depths: shallow (0.6 m) and deep (2 m). Box: Interquartile range; line: median; whiskers: 1.5 × IQR; dots: outliers. Colors denote climate scenario (blue: *Baseline*; red: T + 2 °C; green: P − 10%).

Increasing density raised tree water stress in the shallow soil scenario but had no impact on the deep one. The mean seasonal water-stress coefficient (*K_s_*) was computed as the ratio between *E_p_* and potential transpiration in the absence of water stress (*E_pmax_*). *K_s_* remained unchanged for deep soil but declined with respect to shallow soil (from 0.69 to 0.59; Figure 3). The density effect was smaller than the soil-depth effect, and despite the higher water stress at 408 trees ha^−1^, both yield and water productivity still rose with density (Figure 1 and Figure 4).

### 3.3. Water Productivity

The median *WP_y_* (Figure 4) followed the same trend as median fresh yield (Figure 1). Increasing tree density raised *WP_y_*. Like yield, the median *WP_y_* in the *Baseline* and *P* − 10% scenarios remained similar to each other. The main differences in all sites, soil depths and densities were found for *T* + 2 °C, which showed lower median values. Cordoba and Izmir presented similar ranges, and Pisa presented the lowest *WP_y_*.

**Figure 5:**
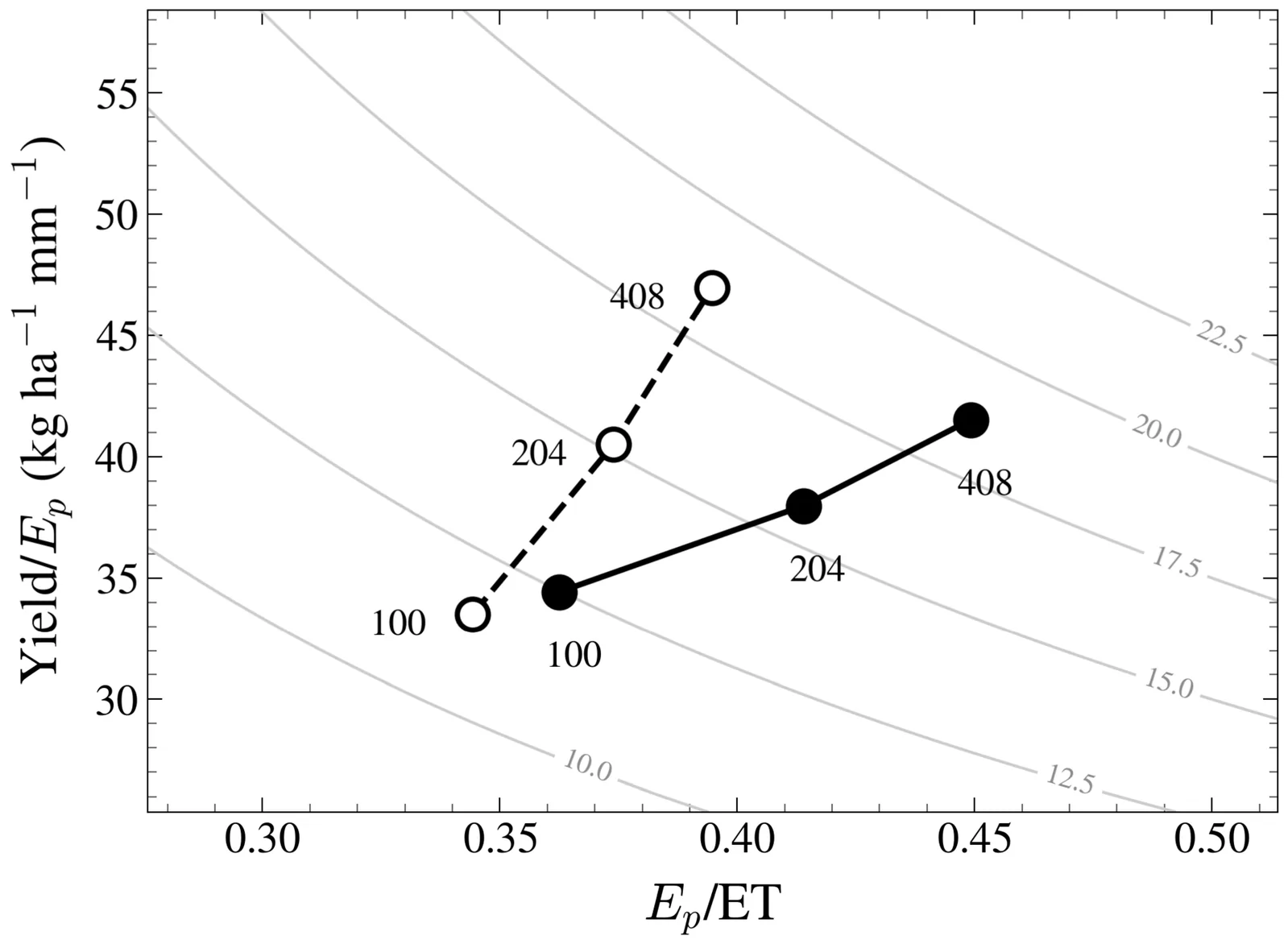
Decomposition of water productivity, *WPy*, into the ratio of yield to transpiration (*E_p_*) and of transpiration to evapotranspiration (*ET*). The grey curves are iso-*WPy* (kg ha^−1^ mm^−1^). Each symbol represents one planting density averaged over the three sites, three climate scenarios and 28 simulated years; labels give the density in trees ha^−1^. Filled versus open symbols denote deep (2.0 m) versus shallow (0.6 m) soil.

Increasing planting density raised *WP_y_* across all simulated combinations, with each trajectory crossing the iso-*WP_y_* curves outward (Figure 5). However, the relative contribution of its two underlying components depended on soil depth. Transpiration efficiency (*TE*, the ratio of yield to *E_p_*) increased with density in both soil types, showing a noticeably steeper response in shallow soil. From 100 to 408 trees ha^−1^, transpiration efficiency rose by 40.2% in shallow soil compared to 20.6% in deep soil. Conversely, the partitioning ratio (*E_p_*/*ET*) increased by 14.6% in shallow soil and 23.9% in deep soil. Thus, the density-driven increase in *WP_y_* under the deep soil scenario resulted from balanced gains in both t *TE* and *E_p_* partitioning. In contrast, the increase in *WP_y_* within the shallow soil scenario was primarily driven by enhanced *TE*.

### 3.4. Stability Analysis

**Figure 6:**
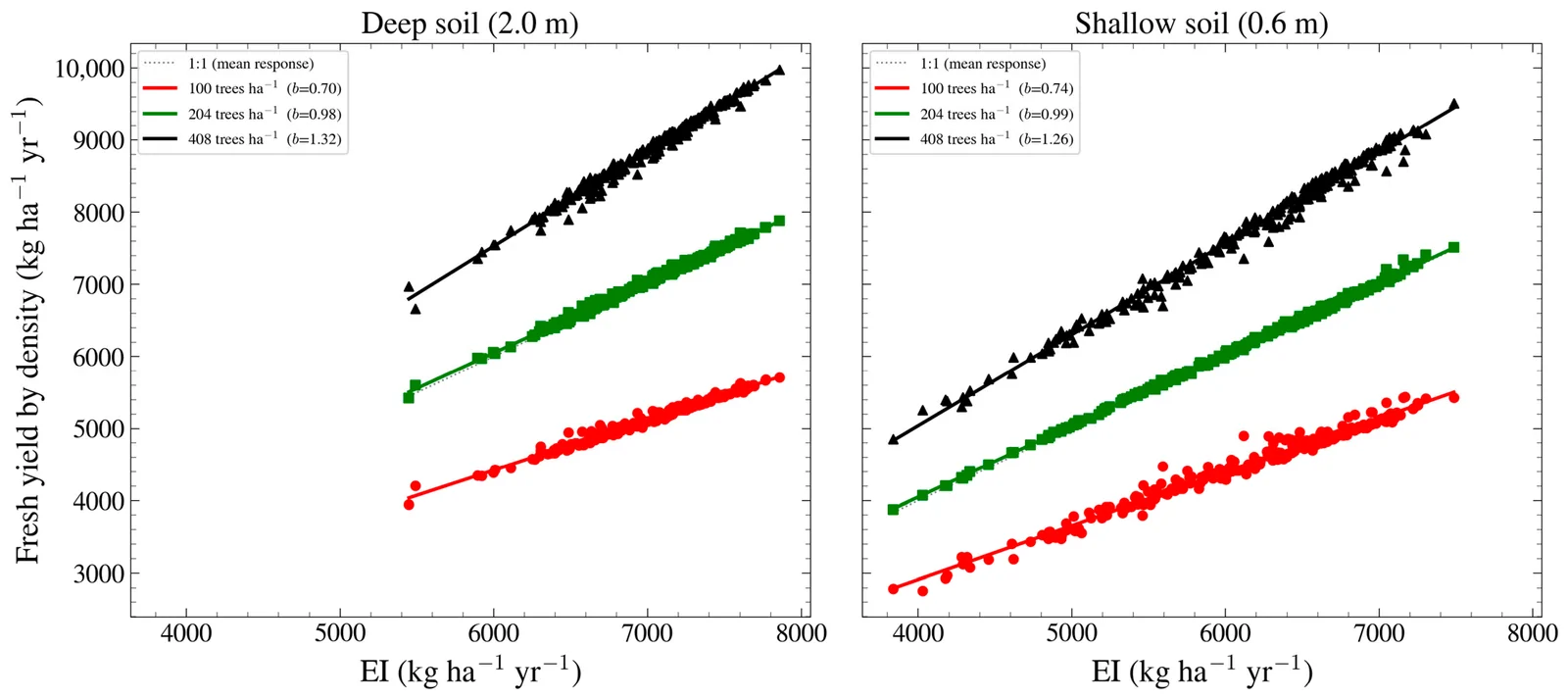
Plots of fresh yield versus environmental index for each of the studied planting densities under deep (2 m) and shallow (0.6 m) soils. The environmental index was calculated as the average fresh yield for each site × year × climatic scenario combination. Marker shape and color indicate planting density (black triangles: 408 trees ha^−1^; green squares: 204 trees ha^−1^ and red circles: 100 trees ha^−1^).

Irrespective of the soil depth scenario, the slope of the fresh yield versus *EI* relationship is above 1 for the simulations with 408 tree ha^−1^, indicating that this density will outperform the other two, especially under deep soils (Figure 6). While the slope in the intermediate planting density (204 tree ha^−1^) is nearly 1, the lowest (100 tree ha^−1^) is below 1, which reveals less yield responsiveness to better or worse environments (i.e., higher stability). Slopes were similar regardless of the soil depth. For instance, the analysis for 204 tree ha^−1^ revealed slopes of 0.98 and 0.99 for deep and shallow soil, respectively. Across densities, the regressions explained almost all variance (R^2^ ≥ 0.98).

## 4. Discussion

### 4.1. Yield and Intensification

Olive production has shifted from low-density, marginal systems toward highly intensified orchards [2]. This intensification has substantially raised olive yields [8], a rise linked primarily to the greater radiation interception of denser canopies [28,29]. The intensification trend has been driven jointly by higher planting densities and the spread of irrigation [2]. Our analysis shows, however, that raising yield through intensification is also achievable under rainfed conditions and that the benefit holds across contrasting weather conditions and soil depths (Figure 1).

Our results agree with Guerfel [9], who reported rising yields when density increased from 51 trees ha^−1^ to 156 trees ha^−1^. Mairech [18] also estimated an 8–20% yield increase when moving from 100 to 204 trees ha^−1^ under rainfed conditions; our results suggest the gain can be somewhat larger. Because Mairech [18] integrated a much wider range of locations, their site-averaged figures may have dampened the response. *Baseline* yields at Córdoba for 204 trees ha^−1^ on deep soil are slightly above the reported values for 277 trees ha^−1^ with similar soil depths in a field experiment in Córdoba [30]. These discrepancies, however, are expected in simulated data, because the model does not include the effect of biotic factors. Therefore, the model simulates the potential attainable yield rather than the actual harvestable yield. Absolute yields, therefore, should be read as indicative rather than site-calibrated, since the model was not validated against measured yields at every site; the robust output is the relative response to density, which is consistent across sites.

Sustained yield gains with intensification rest on two elements: fruit number and the partitioning of the water balance. OliveCan captures water- and heat-driven effects on fruit number and growth [16]. Olive fruit set is well adapted to harsh Mediterranean conditions [31]: once self-thinning ends and fruit number is fixed, only extreme abiotic stress reduces it. Fruit set coincides with the seasonal peak in transpiration (Figure 2) in late spring (April–June), when soil-water reserves are still partly replenished by winter–spring rainfall and evaporative demand is moderate. These conditions can favor a proper fruit set regardless of the tree density analyzed.

The second element is the amount of water routed to transpiration. Regardless of soil depth, as density increased, *E_i_* rises, and *E_s_* and *R* declined. Both *E_s_* and *R* savings are translated into higher soil water availability, which allow increased seasonal *E_p_*. The 408 trees ha^−1^ stand maintains the highest *E_p_* from January to late May, followed by 204 and 100 trees ha^−1^ (Figure 2). A strong proportionality between yield and transpiration is well documented in olive and other semi-arid tree crops [30,32]. After fruit set, *E_p_*, in the 408 stand declines below that of the 204 and 100 stands; this decline, however, coincides with pit hardening, a phenological phase of low sensitivity to water stress that is well exploited in deficit-irrigation programs [33,34].

### 4.2. Yield and Climatic Scenarios

The *P* − 10% scenario produced almost no yield change relative to the *Baseline* (Figure 1). This stems from how the reduction was imposed. The precipitation reduction was imposed for the entire year, not for particular months. In Mediterranean climates, rain falls mainly between autumn and spring, and at all study sites, the seasonal totals tend to exceed the soil water-holding capacity most years—even in the deep-soil scenario—so the surplus is drained. The *P* − 10% scenario therefore trimmed drainage rather than the water available to the trees and hence left *E_p_* largely unchanged (Table 3 and Table 4). Whether a differently distributed reduction—concentrated in summer or larger in magnitude—would erode yield remains to be tested.

Warming negatively affected yields in Córdoba and Izmir (Figure 1). The *T* +2 °C scenario increased *ET*_0_ and, consequently, *E_p_*. Higher *E_p_* supporting the same fruit load intensifies water stress, impairing fruit growth and reducing water productivity (Figure 1 and Figure 4). Furthermore, the actual impact of temperature on fruit set may be more severe than simulated. Temperature increases around 4 °C can induce fruit abortion [35], suggesting that the actual yield penalty from warming could exceed model estimates in Figure 1. Conversely, Pisa showed a positive response to warming. Pisa had the lowest *Baseline* mean temperature, and the *T* + 2 °C scenario elevated its minimum temperatures to levels comparable to those in Córdoba and Izmir while maintaining lower maximum temperatures (Table 2). *T* + 2 °C did not reach the 4 °C thresholds in Pisa. The temperature rise, therefore, enhanced thermal accumulation and vegetative growth.

The climatic scenarios analyzed do not represent a future projection under a specific emission pathway (e.g., an RCP or SSP scenario) but rather a sensitivity analysis for which its sole aim is to broaden the range of environmental conditions explored while holding CO_2_ concentration constant. OliveCan does account for the effects of CO_2_ on photosynthesis and transpiration, a feature that has been exploited in previous analyses with the model [17,18]. However, rising CO_2_ would add a further layer of complexity and could confound the temperature and precipitation signals we set out to isolate. For this reason, we imposed perturbations on two climatic variables—temperature and precipitation—while keeping CO_2_ concentrations unchanged.

### 4.3. Water Productivity and Intensification

*WP_y_* followed the same pattern as yield, rising with planting density at every site and soil depth (Figure 4). The *Baseline* and *P* − 10% scenarios were again almost indistinguishable, whereas the *T* + 2 °C scenario lowered *WP_y_* across the board—consistent with higher evaporative demand and more rapid depletion of soil-water reserves in late spring. Comparable results have been reported in other studies evaluating olive performance under future climate scenarios, where reduced yields driven by more frequent heat events, combined with larger water demands, are identified as the main causes [17,36].

Previous research on irrigated olive orchards has reported water productivity gains with increasing density [7,8]. Under rainfed conditions, however, the *WP_y_* outcome depends on a trade-off that does not arise under irrigation: The yield gain conferred by greater radiation interception must be set against the faster depletion of a finite soil-water store by a larger transpiring stand. The simulations indicate that this trade-off resolved in favor of the denser stands because interception increased faster than transpiration, and interception gains were converted into yield almost without loss (Figure 4 and Figure 5). The ground cover rose by 75% between 100 and 408 trees ha^−1^ at both soil depths (Table 1): against 43% for *E_p_* on deep soil and 23% for shallow soil. Intercepted radiation per unit transpiration increased by 23% and 44%, respectively.

The water sustaining this additional transpiration was released by two terms of the water balance. Averaged over sites and scenarios, increasing density from 100 to 408 trees ha^−1^ reduced *R* by 65 mm and *E_s_* by 35 mm on deep soil (63 mm and 36 mm on shallow), partly offset by a 35 mm (32 mm) increase in *E_i_*; the net gain in *E_p_* was 64 mm on deep soil and 31 mm on shallow soil (Table 3 and Table 4). *E_s_* fell from 52% to 37% for deep soil and from 53% to 41% for shallow soil as density increased, converging towards the 20–40% range reported for olive orchards [11,37]. The higher values simulated at low density are consistent with the large fraction of bare soil exposed at 100 trees ha^−1^. Together, the *E_s_* and *R* shifts redirected a greater share of the water balance towards *E_p_*, which matters because yield responds positively to *E_p_* [30].

### 4.4. Soil Depth Impacts on Yield and WP_y_

Soil depth was a first-order control on yield and *WP_y_*. The deep soil scenario out-yielded shallow ones at every site and density, and it buffered the impact of both *P* − 10% and *T* + 2 °C scenarios (Figure 1 and Figure 4). The larger water-holding capacity of deep soils helps sustain *E_p_* through the summer demand peak, when shallow soils are depleted—the same deep-water buffering that allows trees in well-drained settings to ride out drought [12,13]. Olive is especially well placed to exploit soil depth, developing profuse, plastic root systems that adjust their architecture to maximize water capture [14]. Deep-soil access thus emerges as the principal resilience factor in our analysis: it widens the range of environments over which high density remains advantageous and dampens the yield cost of warming.

Soil depth also played a critical role in the mechanism underlying *WP_y_* gains (Figure 5). Deeper soil supported sustained increases in both *E_p_* and yield with higher tree density. Conversely, in shallow soil, the limited water-holding capacity imposed a stricter constraint on *E_p_* than on yield. Thus, although *WP_y_* gains were similar across both soil depth scenarios, the underlying mechanisms differed. In shallow soil, *WP_y_* gains were primarily driven by higher *TE*, stemming from greater water stress than in the deep soil scenario. The increase in *TE* under stress is a well-documented phenomenon [38,39], as stomatal closure during soil drying reduces water loss proportionally more than carbon assimilation [38].

### 4.5. Stability and Yield

Rainfed planting density has traditionally followed a conservative rationale: allocating a large volume of soil to each tree is assumed to lower the risk of crop failure in dry years by enlarging the per-tree soil-water reservoir. Typical planting densities span roughly 50 to 200 trees ha^−1^, with the upper end being the exception rather than the norm [2].

Yet this caution may be poorly founded. Olive trees tolerate extremely negative water potentials and still produce [30,40]. Moriana [30] recorded more than 5000 kg ha^−1^ from a 277 trees ha^−1^ rainfed stand for which its trees reached leaf water potentials of −8 MPa. The low-risk, wide-spacing strategy therefore looks less like an agronomic optimum than a hedge against the perceived cost of investing land and resources in an orchard that might prove unprofitable. Process-based models offer a way to test that hedge at zero risk.

Our stability analysis bears this out. Figure 6 shows that a density as high as 408 trees ha^−1^ outperforms the other two across a wide range of environments, even on shallow soil. This finding warrants one caveat. As noted in Section 2, the model was run in static mode. Under static mode, the potential fruit number is reset every year. This makes long-term simulations feasible while avoiding large departures from reality, since some tree dynamics—such as root turnover or the response to extreme stress—are still not fully understood [15]. In return, static mode may give higher densities an artificial advantage, because the carry-over effects of stress on the number of fruiting positions available the following year are erased. However, in static mode, the water balance is preserved. It mimics a situation in which the farmers prune the tree every year to keep the canopy volume. Consequently, the density contrast is thereby held clean: the three densities are compared at an identical, defined canopy structure, and what differs between them is the partitioning of the water balance and the climatic conditions experienced.

The slopes reported here may differ from the true ones. We nonetheless expect their ranking to hold. The lowest density—the most stable (slope < 1)—would suit the most marginal, low-yielding environments, but such environments would likely be the exception rather than the rule. On this interpretation, the buffered yield of wide spacing would amount to insurance against a risk that rarely materializes, paid for in forgone yield in almost every year.

The cost–benefit trade-offs of increasing tree density were not analyzed explicitly, as they fall beyond the main objective of this study—namely, to explore the feasibility of higher planting densities under rainfed conditions. We hypothesize, however, that establishment and management costs may cap intensification at a lower density than the biological limit set by tree survival. Field experiments are needed to test their viability under real orchard conditions.

## 5. Conclusions

Olive intensification has carried the industry from marginal dryland production toward high-yielding, well-mechanized systems with clear social and economic benefits—but at the cost of the competitiveness of traditional rainfed farming. Our analysis points to an alternative: Increasing planting density can raise both yield and water productivity under rainfed conditions by increasing radiation interception and reducing soil evaporation and runoff, provided that fruit set is not compromised. The gain is robust to a moderate decline in rainfall but is eroded by warming. Soil depth buffers the interannual yield variability by reducing summer water deficits. Across the studied environments, high densities (408 trees ha^−1^) maximize yield and water productivity. Field experiments testing higher rainfed densities are now desirable to confirm their viability under real orchard conditions.

## Figures and Tables

**Table 1:** Initial orchard characteristics at the start of the simulation on the three sites studied.

Tree Parameters	Tree Densities		
	100 trees ha^−1^	204 trees ha^−1^	408 trees ha^−1^
Ground cover (%)	24	34	42
Leaf area density (m^2^ m^−3^)	1	1	1
Tree volume (m^3^)	61.1	41.1	26.4
Width alley (m)	10	7	7
Distance between trees (m)	10	7	3.5

**Table 2:** Mean climatic conditions at each site and for the three scenarios analyzed. In the *T* + 2 °C scenario, mean temperature is increased by 2 °C with respect to the baseline, while *P* − 10% creates a perturbed climatic series which results from reducing precipitation by 10% every month.

Site	Scenario	T_max_ (°C)	T_min_ (°C)	T_mean_ (°C)	R_s_ (MJ m^−2^ d^−1^)	u_2_ (m s^−1^)	e_a_ (kPa)	P (mm)	ET_0_ (mm)
Córdoba	Baseline	24.2	11.9	18.0	15.2	1.68	1.26	671	1281
	P − 10%	24.3	12.0	18.1	15.2	1.66	1.27	637	1286
	T + 2 °C	26.2	13.9	20.0	15.2	1.68	1.26	671	1411
Izmir	Baseline	22.3	13.3	17.8	16.1	2.89	1.35	786	1403
	P − 10%	22.4	13.4	17.9	16.3	2.91	1.35	641	1410
	T + 2 °C	24.3	15.3	19.8	16.1	2.89	1.34	786	1597
Pisa	Baseline	20.0	9.5	14.8	12.8	1.64	1.22	936	974
	P − 10%	20.1	9.5	14.8	12.7	1.63	1.22	867	971
	T + 2 °C	22.0	11.5	16.8	12.8	1.64	1.22	936	1098

**Table 3:** Mean annual fresh yield and water balance components for rainfed olive orchards simulated with OliveCan across three Mediterranean sites (Córdoba, Izmir, and Pisa), three climate scenarios and three planting densities—**deep soil (2.0 m)**. Values are 28-year means. *E_p_*, tree transpiration; *E_s_*, soil evaporation; *E_i_*, canopy evaporation; *D*, deep drainage; *R*, surface runoff.

Site	Scenario	Density	Yield	Water Balance Components				
				*E_p_*	*E_s_*	*E_i_*	*D*	*R*
		trees ha^−1^	kg ha^−1^	mm yr^−1^				
Córdoba	Baseline	100	5242	154.8	194.9	41.9	171.4	91.4
		204	7188	191.8	177.3	57.9	190.0	38.7
		408	9063	219.9	161.7	71.0	170.3	38.1
	Precipitation − 10%	100	5312	157.4	218.5	42.0	133.4	69.0
		204	7291	195.9	199.3	58.1	141.3	27.2
		408	9196	225.3	181.4	71.3	124.7	27.0
	Temperature + 2 °C	100	5065	162.3	201.7	40.0	161.4	89.8
		204	6945	198.9	184.5	55.3	179.3	38.1
		408	8592	227.0	169.1	68.2	159.1	37.5
Izmir	Baseline	100	5252	147.4	177.1	47.5	236.2	140.8
		204	7219	184.3	161.8	65.8	268.8	63.6
		408	9143	212.8	149.1	80.6	241.2	62.2
	Precipitation − 10%	100	5335	147.6	173.8	40.6	158.9	94.3
		204	7330	184.3	160.4	56.2	171.4	41.1
		408	9292	212.6	148.9	69.0	149.0	40.2
	Temperature + 2 °C	100	5269	155.7	185.6	45.0	224.1	139.2
		204	7247	191.7	171.6	62.1	256.4	63.0
		408	9059	219.1	159.2	76.2	230.3	61.7
Pisa	Baseline	100	4807	132.4	250.0	65.1	333.6	139.9
		204	6601	167.5	226.0	90.5	374.8	61.8
		408	8340	194.8	204.6	111.1	350.3	60.6
	Precipitation − 10%	100	4826	131.8	244.2	62.6	297.4	109.6
		204	6622	166.2	221.2	86.9	325.0	45.5
		408	8355	192.9	200.7	106.6	300.8	44.6
	Temperature + 2 °C	100	4917	147.5	257.7	62.4	315.7	137.8
		204	6758	183.6	235.0	86.4	355.0	60.9
		408	8495	211.1	214.1	105.8	330.8	59.9

**Table 4:** Mean annual fresh yield and water balance components for rainfed olive orchards simulated with OliveCan across three Mediterranean sites (Córdoba, Izmir, and Pisa), three climate scenarios and three planting densities—**shallow soil (0.6 m)**. Values are 28-year means. *E_p_*, tree transpiration; *E_s_*, soil evaporation; *E_i_*, canopy evaporation; *D*, deep drainage; *R*, surface runoff.

Site	Scenario	Density	Yield	Water Balance Components				
				*E_p_*	*E_s_*	*E_i_*	*D*	*R*
		trees ha^−1^	kg ha^−1^	mm yr^−1^				
Córdoba	Baseline	100	4420	132.4	191.9	39.9	210.2	88.6
		204	6053	146.7	174.0	54.9	249.2	37.6
		408	7569	156.7	157.8	67.7	243.5	36.9
	Precipitation − 10%	100	4596	135.4	214.6	40.1	174.6	66.2
		204	6268	151.1	195.3	55.3	203.0	25.9
		408	7828	162.4	177.1	68.2	197.4	25.5
	Temperature + 2 °C	100	3947	141.5	197.8	39.1	198.5	87.0
		204	5481	157.9	179.8	54.0	234.8	36.8
		408	6851	169.6	163.6	66.9	227.5	36.1
Izmir	Baseline	100	4536	118.9	174.6	44.2	297.2	139.1
		204	6166	129.8	159.7	60.5	359.8	63.1
		408	7713	137.9	146.4	74.4	352.5	61.8
	Precipitation − 10%	100	4396	116.3	171.2	38.0	215.8	92.7
		204	5990	126.6	157.7	52.2	256.0	40.5
		408	7487	134.4	145.7	64.3	249.3	39.7
	Temperature + 2 °C	100	3944	128.3	182.2	43.4	283.3	137.1
		204	5441	141.1	167.6	59.8	342.5	62.1
		408	6809	150.8	154.6	73.8	333.5	60.8
Pisa	Baseline	100	4843	140.0	246.9	62.8	348.1	135.9
		204	6656	167.1	223.4	85.7	398.0	59.8
		408	8448	185.2	201.9	104.1	384.3	58.5
	Precipitation − 10%	100	4869	140.2	241.0	60.4	310.1	105.7
		204	6693	166.4	218.1	82.4	347.0	43.6
		408	8499	183.7	197.9	99.8	333.5	42.7
	Temperature + 2 °C	100	4952	155.8	254.1	60.2	329.8	133.7
		204	6769	183.7	231.2	82.5	377.8	58.8
		408	8472	202.5	210.0	100.6	363.5	57.6

## Data Availability

The raw data supporting the conclusions of this article will be made available by the authors on request.
